# Secreted proteins of *Mycobacterium tuberculosis* as therapeutic targets for drug discovery

**DOI:** 10.3389/ftubr.2026.1736139

**Published:** 2026-08-06

**Authors:** Anthony Musaazi Kaweesa, Divya Tiwari

**Affiliations:** Blizard Institute, School of Medicine and Dentistry, Queen Mary University of London, Whitechapel, United Kingdom

**Keywords:** drug discovery, ESX, secretion systems, secreted proteins, tuberculosis, virulence, antivirulence

## Abstract

*Mycobacterium tuberculosis (M. tuberculosis),* the causative agent of tuberculosis secretes proteins during the host-pathogen interaction that facilitate its survival and/or virulence within host cells. Key examples include EsxA and EsxB, CpnT/TNT, phosphatases (SapM, PtpA, PtpB), protein kinase G (PknG), the antigen 85 (ag85) complex, acyl Carrier Protein (AcpM), lipoproteins, and heat shock proteins (Hsps). Together, these secreted factors disrupt host immune responses such as phagosome maturation, antigen presentation, cytokine production, and autophagy thereby aiding in survival of these obligate intracellular bacteria. Several secreted proteins are emerging as therapeutic targets to help meet the need for shorter, more effective, and better tolerated treatment regimens for tuberculosis. Here, we review the current understanding of the major secreted proteins of *M. tuberculosis*. We also discuss progress in the discovery and development of drugs targeting these proteins. Overall, targeting *M.tuberculosis* secreted proteins remains largely in the preclinical stages of drug development. Current limitations include low potency, off-target activity, and redundancy among secreted proteins.

## Introduction

Tuberculosis (TB) is an infectious disease that remains a leading cause of death in both adults and children worldwide, particularly in low- and middle-income countries. TB is caused by *Mycobacterium tuberculosis (M. tuberculosis),* a slow-growing bacterium that primarily infects the lungs. Most individuals infected with *M. tuberculosis* develop latent tuberculosis infection (LTBI) which is asymptomatic. However, around 10% of infected individuals develop active TB disease, which is associated with symptoms such as persistent cough, haemoptysis, fever, weight loss, and night sweats (1, 2). Treatment of active TB involves a combination of multiple antibiotics taken over several months. Although current regimens may potentially cure TB, there are risks of serious adverse effects and adherence to treatment is essential to minimise the development of drug-resistant TB. These challenges underscore the need for novel anti-TB drugs that are more effective and better tolerated.

*M. tuberculosis* secretes an array of proteins that enhance its ability to persist within the host and cause disease. These include surface bound or secreted proteins and lipids with roles such as evasion of host immunity, facilitation of pathogen entry into host cells, or modulation of pro-inflammatory cytokine production. Understanding the roles of these factors is essential for identifying novel therapeutic targets, and for developing better diagnostic tests and novel vaccine strategies. Here, we review the major secreted proteins of *M. tuberculosis* with a focus on their potential as therapeutic targets (summarized in Table 1). Many of these proteins are immunodominant antigens and are of active interest in the field of vaccine development (3), however, their roles as therapeutic targets are under evaluated. Many classic TB drugs require access to the cytosol of *M. tuberculosis,* although this approach is limited by the robust structure and high impermeability of the mycobacterial cell wall. Furthermore, drugs that reach the cytosol may still be inhibited by xenobiotic mechanisms or efflux pumps. Targeting secreted proteins is therefore an attractive approach as it does not necessarily require penetration of the cell wall and may, in principle, bypass the associated mechanisms of drug resistance.

**Table 1:** Summary of secreted virulence proteins of *Mycobacterium tuberculosis* and inhibitors directly targeting them.

Secreted protein(s)	Main roles in pathogenesis	Reported inhibitors/modulators	References
EsxA and EsxB	Phagosome rupture and cytosolic escape; modulates host immune responses via TLR2 interaction.	IMB-BZ	(17, 20, 25, 26)
		HCL2	
		Ethoxzolamide (indirect; reduces ESX-1 secretion via PhoPR downregulation)	
Ag85A, Ag85B, Ag85C	Attachment and invasion of *M. tuberculosis;* Mycolyl-transferase activity maintains structural integrity of mycobacterial cell envelope.	Ebselen and ebselen derivatives	(29, 32, 39)
		I3-AG85	
		Cyclophostin and cyclipostins (CyC) analogues	
CpnT/TNT	Necrotic cell death; nutrient uptake.	No direct inhibitors reported yet.	(45)
SapM	Inhibits phagosome maturation and phagosome-lysosome fusion.	2-phospho-L-ascorbic (2P-AC)	(48, 50, 51)
		Tyrphostin 51	
		Tyrphostin Ag183	
PtpA	Inhibits phagosome maturation and acidification.	Stevastelins	(59–64)
		Roseophilins	
		Prodigiosins	
		Hydroxypyrrol benzoic acids	
		Chalcones	
		L335-M34	
		Aryl DFMP	
PtpB	Inhibits the expression of pro-inflammatory mediators and supresses apoptosis.	I-A09	(63, 66–68)
		L01Z08	
		Kuwanon G	
		Fusarielin M	
PknG	Inhibits phagosome-lysosome fusion.	AX20017	(72, 75–79)
		AZD7762	
		R406	
		NU-6027	
		Sclerotiorin	
		RO9021	
		Flavanones	
AcpM	Inhibits apoptosis, ROS production, phagosomal maturation, and autophagy.	No direct inhibitors reported yet.	(80–83)
	Disrupts JNK signaling.		
Lipoproteins	LprA and LpqH induce TNF and IL-12 production via TLR-2 agonism	No direct inhibitors reported yet.	(84, 85)
Heat Shock Proteins (Hsps)	Enable adaptation to intracellular stressors such as oxidative stress, hypoxia, and nutrient deprivation.	IgA 2E9 (targets HspX)	(95, 103)
		Telaprevir (targets Hsp70)	

Ag85, antigen 85; CpnT, Channel protein with necrosis-inducing toxin; TNT, tuberculosis necrotizing toxin; SapM, secreted acid phosphatase M; PtpA/B, protein tyrosine phosphatase A/B; PknG, protein kinase G; AcpM, acyl carrier protein; TLR2, toll-like receptor 2; ROS, reactive oxygen species; JNK, c-Jun N-terminal kinase; TNF, tumor necrosis factor; IL-12, interleukin 12.

This review focuses on secreted *M. tuberculosis* proteins as therapeutic targets, rather than on the medicinal chemistry or pharmacokinetic properties of drug candidates. We discuss the biological and pathogenic properties of these proteins and summarise progress towards their therapeutic targeting.

## Pathogenesis of *Mycobacterium tuberculosis—*an overview

Transmitted via aerosol droplets, *M. tuberculosis* enters the lungs where it is subsequently phagocytosed, primarily by alveolar macrophages. After the uptake of particles by phagocytic cells, the newly formed phagosome normally undergoes a maturation process whereby its luminal pH gradually decreases before fusing with lysosomes to obtain degradative enzymes (4). However, through various mechanisms, *M. tuberculosis* can evade the hostile intracellular environment and persist within host macrophages. In response, macrophages and other immune cells form an organised collection around infected macrophages called a granuloma, which helps contain the infection. Most infected individuals develop latent tuberculosis infection (LTBI) where *M. tuberculosis* remains in an asymptomatic state of dormancy, potentially for decades (5). In contrast, 5%–10% of infected individuals develop active TB disease in which *M. tuberculosis* reactivates, continues to replicate, and causes significant tissue pathology (1, 2). *M. tuberculosis* may eventually escape from the granuloma, spread to the lung interstitium, and potentially to extrapulmonary sites. Extrapulmonary TB most commonly involves the lymph nodes and but may also affect the brain, abdomen, genitourinary system, and most other sites in the body (6).

## Overview of protein secretion systems in *Mycobacterium tuberculosis*

In *M. tuberculosis*, secreted proteins are exported by secretion systems composed of multiple protein components that recognise and transport substrates across the cytoplasmic membrane. The major secretion systems of *M. tuberculosis* are the ESX, or Type VII secretion systems, the general secretory (Sec) pathway, and the twin-arginine translocation (Tat) pathway. Each secretion system differs in its structure and associated substrates (7).

The ESX secretion systems comprise five type VII secretion systems, ESX-1 to ESX-5, each encoded by a distinct ESX locus in the *M. tuberculosis* genome. ESX-1 is the most extensively characterised and is responsible for the secretion of a stable heterodimer composed of EsxA (also known as 6-kDa early secretory antigenic target, or ESAT-6) and EsxB (also known as Culture Filtrate Protein-10, or CFP-10) (8). Other ESX-1 substrates include members of the Esp family, such EspA, EspB, and EspC, which contribute to ESX-1-dependent secretion and virulence.

The ESX-1 translocation machinery is composed of ESX-conserved components (Ecc proteins), which form the core secretion apparatus and interact with ESX-1 substrates during export. These include membrane-associated proteins and cytosolic ATPases, such as EccCa1 and EccCb1 (7, 9, 10). ESX-1 is encoded by the region of difference 1 (RD1) gene locus which is absent from all strains of the Bacillus Calmette–Guérin (BCG) vaccine. The loss of RD1 is therefore thought to have contributed to the attenuation of *Mycobacterium bovis* during the development of BCG, highlighting the importance of ESX-1 in mycobacterial virulence (11). Less is known about the other ESX secretion systems. ESX-3 and ESX-5 have also been implicated in *M. tuberculosis* virulence (12, 13), whereas the roles of ESX-2 and ESX-4 remain poorly characterised.

**Figure 1:**
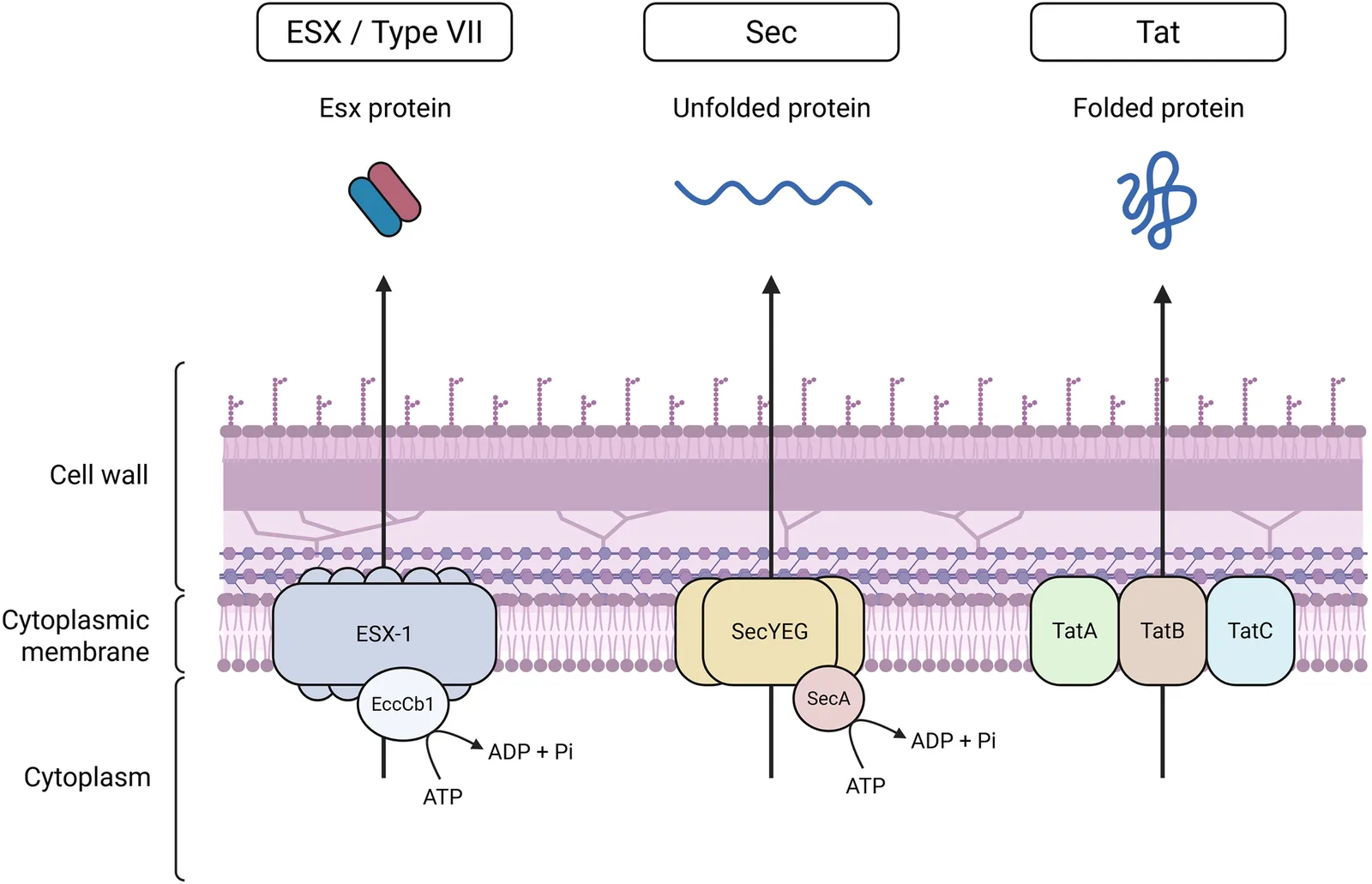
Overview of the major protein secretion systems in *Mycobacterium tuber*culosis. The ESX or type VII pathway exports Esx substrates driven by ATP hydrolysis from ATPases such as EccCb1. Other ATPases have been omitted for simplicity. The Sec pathway exports predominantly unfolded proteins through the SecYEG channel driven by ATP hydrolysis from SecA1 and SecA2 ATPases (represented as SecA). The twin-arginine translocation (Tat) pathway exports folded proteins through the TatABC complex. The structures shown here are simplified and not drawn to scale. Created in BioRender. Kaweesa, A. (2026) https://BioRender.com/vzo84w8.

Unlike the ESX secretion systems, the Sec and Tat pathways are not specific to mycobacteria. The Sec pathway is highly conserved and mainly comprises two ATPases, named SecA1 and SecA2, which are associated with a transmembrane protein-conducting channel named SecYEG. The Sec pathway typically exports unfolded proteins with an N-terminal Sec signal sequence. In contrast, the Tat pathway consists of three transmembrane proteins named TatA, TatB, and TatC, and typically secretes folded proteins with an N-terminal Tat signal sequence (8) The differences between these secretory systems are highlighted in Figure 1.

## EsxA and EsxB

EsxA and EsxB are small helical proteins with molecular weights of 6 kDa and 10 kDa, respectively. As previously mentioned, they are secreted together as a stable heterodimer via the ESX-1 secretion system (14, 15) and are both essential for virulence (16). EsxA has been shown to dissociate from EsxB under acidic conditions and form a transmembrane structure with membrane-permeabilising activity (MPA). EsxA has therefore been implicated in mediating phagosome rupture within host cells and facilitating the translocation of *M. tuberculosis* into the host cytosol (15, 17–19). EsxA also interferes with normal macrophage function by interacting with toll-like receptor 2 (TLR2) to inhibit the production of pro-inflammatory cytokines, and by upregulating the expression of caspases to promote apoptosis (20, 21). Less is known about the role of EsxB and unlike EsxA, it is not known to have significant MPA. However, it is important for the secretion and stability of EsxA, and interacts with the EccCb1 ATPase (22, 23).

Multiple strategies for directly targeting EsxA/B have been proposed. One approach is to disrupt protein-protein interactions including the binding of EsxA to TLR2, the association of EsxB with EccCb1, or the EsxA-EsxB heterodimer. Protein-protein interactions are known to be difficult to disrupt selectively with conventional small molecules (24). Still, a small molecule named IMB-BZ which was reported to inhibit the secretion of EsxB by blocking its interaction with EccCb1. In *M. marinum* infection models, IMB-BZ reduced bacterial cytolytic activity to a level comparable with an ESX-1 deficient strain, countered ESX-1-mediated defects in phagosome maturation, and reduced survival of *M. marinum* in THP-1 macrophages with minimal cytotoxicity (25). Disruption of protein-protein interactions has also been reported with a few other inhibitor types. HCL2 is a peptide derived from ﻿human cytochrome c oxidase subunit 3 (COX3) and attenuates virulence by binding EsxA and disrupting the EsxA-EsxB complex (26). More recently, Bates et al. showed that cytoplasmic expression of an antibody-derived ESAT-6-specific nanobody successfully blocked *M.tuberculosis* growth in macrophages (27). Together, these studies provide proof-of-concept for targeting the EsxA/B axis directly.

Aside from disrupting protein-protein interactions, another strategy is to prevent EsxA from interacting with lipid membranes, hence reducing its membrane-permeabilising activity. However, this would need to avoid disruption of host cell membrane processes and pharmacological inhibitors that specifically block this mechanism have not yet been reported (24).

Lastly, instead of targeting the EsxA and EsxB proteins themselves, some molecules work indirectly by targeting the ESX-1 translocation machinery, or by altering its regulation. This aims to reduce the secretion of ESX-1 substrates in general, overcoming the difficulties of directly targeting the small proteins. One example is ethoxzolamide (ETZ) which is an FDA-approved carbonic anhydrase inhibitor used clinically as a diuretic and for the treatment of glaucoma. Through an unclear mechanism, it leads to downregulation of the PhoPR regulon of *M.tuberculosis* which modulates ESX-1-mediated secretion by sensing and responding to acidic pH within macrophages. Treatment of *M.tuberculosis* with ETZ resulted in a phenotype comparable to the highly attenuated PhoPR mutant strain and inhibited bacterial growth in macrophages and mice (28).

## Antigen 85 (Ag85) complex

The extracytoplasmic proteins that make up the antigen 85 (Ag85) complex are also known as mycobacterial fibronectin-binding proteins. The complex includes the homologous proteins Ag85A, Ag85B, and Ag85C which are each encoded by distinct *fbp* genes and are mostly secreted in a 2:3:1 ratio, respectively, via the Sec pathway (7, 29–31). The fibronectin-binding properties of the Ag85 complex are thought to facilitate the attachment and invasion of *M. tuberculosis* to host cells (32). Furthermore, the Ag85 complex contributes to the assembly and maintenance of the mycobacterial cell envelope. It exhibits mycolyl-transferase activity which catalyses the attachment of mycolic acid to arabinogalactan, a major polysaccharide that contributes to the structural integrity of the mycobacterial cell envelope. The mycolyl-transferase activity also catalyses the biosynthesis of trehalose dimycolate (TDM), also known as cord factor, which is a major glycolipid responsible for the cord-like microscopic appearance of virulent *M. tuberculosis* colonies (29, 33).

A key challenge in targeting the Ag85 complex is functional redundancy between its isoforms. Despite this, the active site topology of Ag85A, Ag85B and Ag85C is highly conserved, which provides a rationale for developing inhibitors with activity against multiple isoforms (34, 35). Doing so may also raise the mutational threshold for resistance, as mutation of a single *fbp* gene may be insufficient to confer resistance to such inhibitors.

The development of Ag85 inhibitors has focused largely on the use of small molecules to disrupt mycolyltransferase activity by targeting active sites or allosteric sites. An early class was the trehalose analogues which were designed to act at the active site by mimicking trehalose-containing substrates (34, 35). The early trehalose analogues provided proof-of-concept for active site inhibition but generally showed limited potency and whole-cell activity (34, 36, 37). Despite this, given the role of Ag85 in cell wall integrity, even partial inhibition of its activity could be sufficient to sensitise the bacterium to antibiotics or other cell wall stressors (38). A further Ag85 inhibitor, I3-A85, has also been reported and faces similar limitations in potency (39).

Ag85 inhibitor development then expanded beyond standard substrate-mimetic approaches. Ebselen and its derivatives act as allosteric inhibitors through covalent modification of Ag85C at Cys209, with ebselen showing additional activity against Ag85A and Ag85B. In contrast, the cyclophostin and cyclipostins (CyC) analogues are covalent active site inhibitors with some members targeting the catalytic Ser124 of Ag85C. Both classes of compounds have been shown to inhibit the growth of extracellularly replicating *M. tuberculosis.* The activity of ebselen and its derivatives requires validation in macrophage infection models, whereas the CyC analogues have been reported to inhibit the intracellular growth of *M. tuberculosis* with limited host-cell cytotoxicity (40–42).

Recently, a novel antibody-based approach using nanobodies has been proposed. Compared to conventional antibodies, nanobodies are smaller, single-domain antibody fragments that may access poorly accessible epitopes more readily. In principle, they may disrupt Ag85 by restricting access to the active site, limiting Ag85-cell-envelope interactions, or stabilising non-productive enzyme conformations. However, there remains a lack of experimental evidence for the functional effects of this approach under physiological conditions (38).

## CpnT and TNT

Channel protein with necrosis-inducing toxin (CpnT) is located on the outer membrane of *M. tuberculosis* (43). The Secretion of CpnT depends mainly on ESX-5, although functional ESX-1 and ESX-4 systems are also required (44). The C-terminal domain of CpnT functions as a cleaved toxin and was therefore named the tuberculosis necrotizing toxin (TNT). TNT is an NAD^+^ glycohydrolase and was found to hydrolyse NAD^+^ *in vitro,* resulting in necrotic cell death of infected macrophages (45). CpnT also facilitates the uptake of nutrients to support the survival of *M. tuberculosis*. CpnT mutants of *M.bovis* BCG *and M.* tuberculosis showed reduced growth relative to wild-type strains when glycerol was provided as a sole carbon source. Indeed, glycerol uptake was minimal in the CpnT mutant of *M.bovis* BCG but rapid in the wild type strain (46).

There have been no reports of direct drug-like CpnT/TNT inhibitors to date, although the dependence of CpnT on ESX-1 and ESX-4 for secretion and cytosolic delivery indicates that the pathway is indirectly targetable. An alternative approach could exploit IFT, an endogenous antitoxin produced by *M. tuberculosis* to protect itself from the necrotising effects of TNT. The discovery and development of molecules that mimic IFT would be required for this unique approach (45).

## SapM

Secreted acid phosphatase M (SapM) is a 28-kDa lipid phosphatase that has been linked to the SecA2 secretion pathway (47). SapM inhibits phagosome maturation and phagosome–lysosome fusion in host cells by preferentially dephosphorylating phosphatidylinositol 3-phosphate (PI3P), a key regulator of lysosomal trafficking, thereby reducing PI3P levels on the phagosomal membrane (48). SapM mutants of *M. tuberculosis* failed to arrest phagosome maturation and showed reduced growth in THP-1 macrophages and guinea pig tissues (49). Likewise, phagosomes of infected macrophages treated with sodium molybdate, a non-selective acid phosphatase inhibitor, showed enhanced maturation and higher levels PI3P on their membranes compared to non-treated counterparts, indicating reduced SapM activity (48).

The development of SapM inhibitors has largely focused on blocking its phosphatase activity through competitive or uncompetitive inhibition. One competitive inhibitor is 2-phospho-L-ascorbic (2P-AC) which is a stable vitamin C (ascorbic acid) derivative. However, it demonstrated limited potency in macrophage infections (50). More recently, additional selective inhibitors of SapM with improved potency were identified. Unlike 2P-AC, these novel inhibitors are uncompetitive, enabling sustained inhibition at high substrate concentrations. The best among them were Tyrphostin 51 with an IC_50_ of 6.3 µM, and Tyrphostin Ag183 with an IC_50_ of 8.2 µM. Both compounds share a trihydroxy-benzene moiety, which likely contributes to their potency (51).

## PtpA and PtpB

Mycoabcterial PtpA and PtpB are secreted protein tyrosine phosphatases (PTPs). PtpA inhibits phagosome maturation and acidification. Host substrates of PtpA include vacuolar protein sorting 33B (VPS33B) which functions in endolysosomal trafficking, and vacuolar H^+^-ATPase (V-ATPase) which functions in phagosome acidification (52–54). PtpA also suppresses apoptosis and the innate immune response by inhibiting c-Jun N-terminal kinase (JNK) and p38 mitogen-activated protein kinase (MAPK) signaling (55, 56). In contrast, PtpB inhibits the expression of the pro-inflammatory cytokines tumour necrosis factor (TNF), interleukin-1β (IL-1β), and interleukin-6 (IL-6) (57, 58).

PtpA has high structural similarity to human PTPs such as PTP1B. As a result, many of the studied small-molecule inhibitors lack specificity to PtpA. This includes the ﻿stevastelins, roseophilins, prodigiosins, ﻿hydroxypyrrol benzoic acids, and chalcones (59–61). Nonetheless, a small number chalcones with IC_50_ values in the low micromolar range were particularly selective and were shown to inhibit the intracellular growth of *M. tuberculosis* (62). Other notable inhibitors of PtpA include L335-M34 and members of the aryl difluoromethylphosphonic acid (DFMP) class (63, 64).

In contrast, PtpB has low structural similarity with human PTP1B and is therefore a more attractive drug target. Numerous inhibitors of PtpB have been discovered, and have been reviewed in detail by Alsayed et al. (65). Notable examples include I-A09, L01Z08, Kuwanon G, and Fusarielin M, which have all been shown to reduce the bacterial burden in macrophages infected with either *M. tuberculosis* or *Mycobacterium bovis* (*M.bovis)* BCG (63, 66–68).

## PknG

Protein kinase G (PknG) is an 82 kDa serine/threonine kinase associated with pathogenic bacteria including *M. tuberculosis*. The kinase domain of PknG is flanked by crucial N-terminal and C-terminal domains that regulate its kinase activity (69, 70). The secretion of PknG has been linked to the SecA2 pathway, despite the protein lacking a sec signal peptide (71). Functionally, PknG has been implicated in the inhibition of phagosome–lysosome fusion and in promoting *M. tuberculosis* survival under stress and hypoxic conditions (72, 73).

A challenge of therapeutically targeting mycobacterial PknG is its high homology to eukaryotic serine/threonine kinases. Off-target effects could therefore impair host signal transduction pathways and compromise host cell viability. The particular focus of this approach has been on identifying inhibitors with high selectivity for mycobacterial PknG. Early studies identified AX20017 as potent inhibitor of PknG with high selectivity for mycobacterial PknG relative to 28 archetypal human kinases, including protein kinase Cα. The high selectivity of AX20017 for PknG can be attributed to the unique amino acid side chains within its narrow binding pocket that are absent in human kinases, which suggests that AX20017 may have relatively few off-target effects within host cells (72, 74). When murine macrophages infected with *Mycobacterium bovis* were treated with AX20017, lysosomal localisation of the bacterium increased in a dose-dependent manner. The compound also reduced the intracellular growth of *M.bovis* and *M. tuberculosis* murine macrophages at 0.1–10 *μ*M (72).

Other PknG inhibitors were later identified, notably AZD7762, R406, NU-6027, and sclerotiorin. Each compound was shown to inhibit the growth of intracellular mycobacteria, but AZD7762 was mildly cytotoxic to macrophages, suggesting mild off-target activity (75–77). More recently identified inhibitors of PknG include RO9021 and the natural flavanones. RO9021 was found to have inhibitory activity that was comparable to AX20017 (78), and six flavanones demonstrated high binding affinity to PknG (79). However, the efficacy of RO9021 and the flavanones require validation in macrophage infection models.

## AcpM

Acyl Carrier Protein (AcpM) is an important enzyme of the FAS-II enzyme system for fatty acid biosynthesis in *M. tuberculosis*. It is involved in loading long chain meromycolic acids during fatty acid elongation (80). AcpM is secreted in macrophages during infection and inhibits apoptosis, ROS production and interferes with activation of the JNK signaling pathway (81). AcpM prevents phagosomal maturation and inhibit autophagy by upregulating miRNA-155-5p expression (82, 83). Given its dual role in Mycobacterial cell wall biosynthesis and host immune modulation, AcpM is an attractive drug target with multi-modal mechanism of action.

## Lipoproteins

*M. tuberculosis* lipoproteins play multifaceted roles in virulence because of their involvement in many cellular functions that are important for bacterial survival, including cell wall assembly, lipid transport as well as immune modulation via TLR2 signaling.

LprA and LpqH are the most well studied lipoproteins that are TLR-2 agonists that activate myeloid differentiation primary response 88 (MyD88)-dependent signaling thereby inducing TNF and interleukin-12 (IL-12) production from macrophages (84, 85). LprE is crucial for *M. tuberculosis* virulence as it regulates expression of cathelicidin, an antimicrobial peptide, via TLR2-dependent p38-MAPK-CYP27B1-VDR signaling pathway (86). LpqN was shown to interact with human Casitas B-lineage lymphoma (CBL) E3 ubiquitin ligase, which is required for bacterial survival, however the mechanism involved in unclear (87). In bacteria, LpqN directly binds trehalose monomycolate, the Mycobacterial membrane protein large 3 (MmpL3) and Ag85A substrate, and may a play a role in their transport to cell wall (88). LprG and LppX are other important lipoproteins with roles in bacterial physiology, with implications on host-pathogen interaction. LprG functions in cell wall homeostasis by binding and transporting lipoarabinomannan and triacylated *M. tuberculosis* glycolipids to the bacterial cell surface (89, 90) while LppX facilitates phthiocerol dimycocerosates (PDIM) translocation to the bacterial surface (91).

Lipoproteins are non-redundant, immunogenic, and often essential for intracellular survival. Inhibiting lipoprotein maturation or export could disrupt lipid homeostasis that can compromise bacterial viability. In addition, a compromise in their role in modulating host immune response favouring intracellular bacterial survival may aid in bacterial clearance. This dual role of *M. tuberculosis* lipoproteins in pathogenesis and immune recognition positions them as key focal points in next-generation strategies for shorter, more effective TB chemotherapy and improved immunotherapeutic interventions.

## Heat shock proteins

Secreted heat shock proteins (HSPs) are molecular chaperones that play crucial roles in bacterial virulence, stress adaptation, and immune modulation. Various HSPs present in *M. tuberculosis* are involved in protein folding and protection in various stress conditions that *M. tuberculosis* faces due to its intracellular lifestyle, such as oxidative stress, hypoxia, and nutrient deprivation. They help *M. tuberculosis* survive during dormancy and reactivation phases by maintaining proteostasis, thereby contributing to long-term persistence in the host. HSPs like HspX (Hsp16.3/Acr/Rv2031c), Hsp65 (GroEL2/Rv0440), Hsp70 (DnaK/Rv0350), and Hsp90 (Rv2299c) are secreted in host macrophages upon infection and modulate the host immune response.

HspX is one of the most abundant proteins expressed during TB infection and is part of the DosR (dormancy survival regulator) regulon, that is regulated under hypoxic, nutrient-deprived, and nitric oxide stress conditions (92, 93). HspX drives M2 polarization in macrophages resulting in increased production of IL-10, TGF*β* etc (94). A human monoclonal IgA antibody against hspX, known as IgA 2E9, was shown to reduce lung bacterial load, involving enhanced phagocytosis (95, 96).

Hsp65 is essential for bacterial cell wall integrity and stress tolerance. It is also a potent antigen that stimulates both CD4⁺ and CD8⁺ T-cell responses (97). Hsp65 induces macrophage activation through TLR4 signaling, leading to the release of pro-inflammatory cytokines which play an important role in granuloma formation and bacterial containment (98).

Hsp70 (DnaK/Rv0350) is a key molecular chaperone involved in the refolding of denatured proteins and prevention of protein aggregation under stress. Secreted Hsp70 interacts with TLR2, TLR4, and CD40 receptors on macrophages and dendritic cells, triggering cytokine production and enhancing antigen presentation (97, 99, 100). Hsp70 is robust immunodominant T-cell antigens (101). Because of its immunogenic nature and surface accessibility, Hsp70 is being explored as both a vaccine antigen and a target for small-molecule inhibitors aimed at disrupting *M. tuberculosis* stress tolerance. One example is telaprevir, a repurposed small molecule inhibitor that was shown to enhance the efficacy of aminoglycosides against mycobacterial cells, and reduce their resistance to rifampicin (102, 103).

Hsp90 (Rv2299c) has attracted attention as a potent anti-TB vaccine candidate and anti-tumor potential. Mice injected with purified Rv2299c exhibited an increased proportion of CD4^+^ T cells and CD8^+^ T cells among splenocytes (104, 105).

The role of HSPs as molecular sentinels, preserving bacterial proteome stability and their strong antigenicity make them attractive drug and vaccine targets. Small-molecule inhibitors of bacterial Hsp70 have shown bactericidal synergy with existing TB drugs by destabilizing stress response networks which is a promising approach for further drug development.

## Conclusions and future perspectives

The secreted proteins of *M. tuberculosis* vary in structure, function and secretory mechanism. Some have roles in virulence, including phagosome rupture and immune evasion, whereas others support homeostatic processes such as cell wall assembly and maintenance. Secreted proteins may, in principle, serve as more accessible drug targets and provide a means to attenuate virulence or sensitise *M. tuberculosis* to standard antitubercular drugs. Compared with bactericidal approaches, targeting secreted proteins that function as virulence factors may also reduce selective pressure for antibiotic resistance by attenuating virulence rather than directly inhibiting bacterial growth.

The unique properties of each secreted protein will need to be considered when designing and optimising therapeutic strategies. For proteins with defined enzymatic functions such as the Ag85 proteins, catalytic or allosteric inhibition with small molecules may be appropriate. By contrast, non-enzymatic proteins such as EsxA and EsxB may require alternative approaches including disruption of protein-protein interactions, blockade of secretion systems, peptide mimetics, or antibody-based strategies.

Several challenges remain in targeting secreted *M.tuberculosis* proteins. From a biological perspective, these include limited potency of many candidate drugs, off-target activity, and functional redundancy among some secreted proteins. Some secreted proteins are also immunodominant antigens or innate immune receptor agonists, meaning that their inhibition may compromise protective host immune responses. From a translational perspective, candidate discovery is slowed by a lack of suitable screening assays since standard *in vitro* growth inhibition screens are of limited use for targets that contribute primarily to virulence rather than *in vitro* growth (106). One potential solution is to prioritise secreted proteins that also play an important role in bacterial survival; for example, the Antigen 85 complex which is an immunodominant T-cell antigen but also required for TDM biosynthesis, a lipid critical for bacterial survival (39, 107, 108). Indeed, Ebselen (40), a specific competitive inhibitor of Ag85C with antimycobacterial activity has been demonstrated to potentiate the activity of streptomycin (109). Such targets may retain compatibility with conventional growth-based screening assays while preserving some of the advantages of targeting secreted proteins, including target accessibility to inhibitors and a reduced need for intracellular drug accumulation.

Furthermore, pharmacokinetic optimisation, delivery to infected tissues and macrophage compartments, and integration with existing TB drug regimens will need to be considered. Future studies should therefore prioritise demonstration of target vulnerability during infection and explore whether inhibition produces a meaningful biological benefit that would justify further development of this therapeutic approach. Similar principles are being explored in *M. abscessus* where CRISPRi-mediated gene silencing has been used to prioritise targets that are essential for bacterial growth and remain vulnerable during infection (110). Although this work did not focus on secreted proteins, it highlights a potential route to mitigating the issue of poor *in vivo* efficacy.

These are exciting times in the field of TB drug discovery when multiple new drugs have advanced through TB drug development pipeline and clinical trials are being designed with all new drug regimens. This is an opportune moment to focus on targeting *M. tuberculosis* secreted proteins and virulence factors for use as adjuncts to standard chemotherapy. Further research into their biological role and therapeutic potential will shape the pathway for their application in the field of TB drug development.
